# Insertion sequence contributes to the evolution and environmental adaptation of *Acidithiobacillus*

**DOI:** 10.1186/s12864-023-09372-8

**Published:** 2023-05-25

**Authors:** Shanshan Huang, Huiying Li, Liyuan Ma, Rui Liu, Yiran Li, Hongmei Wang, Xiaolu Lu, Xinping Huang, Xinhong Wu, Xueduan Liu

**Affiliations:** 1 School of Minerals Processing and Bioengineering, Central South University, 410083 Changsha, China; 2 Hubei Key Laboratory of Yangtze Catchment Environmental Aquatic Science, School of Environmental Studies, China University of Geosciences, 430074 Wuhan, China; 3 State Key Laboratory of Biogeology and Environmental Geology, China University of Geosciences, 430074 Wuhan, China

**Keywords:** Insertion sequence, *Acidithiobacillus*, Genome, Evolution, Environmental adaptation

## Abstract

**Background:**

The genus *Acidithiobacillus* has been widely concerned due to its superior survival and oxidation ability in acid mine drainage (AMD). However, the contribution of insertion sequence (IS) to their biological evolution and environmental adaptation is very limited. ISs are the simplest kinds of mobile genetic elements (MGEs), capable of interrupting genes, operons, or regulating the expression of genes through transposition activity. ISs could be classified into different families with their own members, possessing different copies.

**Results:**

In this study, the distribution and evolution of ISs, as well as the functions of the genes around ISs in 36 *Acidithiobacillus* genomes, were analyzed. The results showed that 248 members belonging to 23 IS families with a total of 10,652 copies were identified within the target genomes. The IS families and copy numbers among each species were significantly different, indicating that the IS distribution of *Acidithiobacillus* were not even. *A. ferrooxidans* had 166 IS members, which may develop more gene transposition strategies compared with other *Acidithiobacillus* spp. What’s more, *A. thiooxidans* harbored the most IS copies, suggesting that their ISs were the most active and more likely to transpose. The ISs clustered in the phylogenetic tree approximately according to the family, which were mostly different from the evolutionary trends of their host genomes. Thus, it was suggested that the recent activity of ISs of *Acidithiobacillus* was not only determined by their genetic characteristics, but related with the environmental pressure. In addition, many ISs especially Tn3 and IS110 families were inserted around the regions whose functions were As/Hg/Cu/Co/Zn/Cd translocation and sulfur oxidation, implying that ISs could improve the adaptive capacities of *Acidithiobacillus* to the extremely acidic environment by enhancing their resistance to heavy metals and utilization of sulfur.

**Conclusions:**

This study provided the genomic evidence for the contribution of IS to evolution and adaptation of *Acidithiobacillus*, opening novel sights into the genome plasticity of those acidophiles.

**Supplementary Information:**

The online version contains supplementary material available at 10.1186/s12864-023-09372-8.

## Background

Acid mine drainage (AMD) is a unique aquatic habitat formed by the interactions between sulfide minerals with water, atmosphere and microorganisms in mining area. It is typically characterized by low pH, oligotrophy and excessive heavy metals such as copper, lead, zinc and cadmium as well as sulfate ions [1, 2]. Even in this extremely acidic environment, a variety of acidophilic microorganisms still survive and thrive, such as *Acidithiobacillus*, *Leptospirillum* and *Sulfobacillus* [3, 4]. Their ecological adaptation and evolution based on genome researches have attracted much attention in recent years [5–7].

The bacteria in genus *Acidithiobacillus* serve as the representatives for the investigation of microbial survival mechanisms and applications in the AMD environment. *Acidithiobacillus* spp. are capable of oxidizing reduced sulfur (*A. thiooxidans* and *A. caldus*), and some could also catalyze the oxidation of ferrous iron (*A. ferrooxidans*, *A. ferrivorans* and *A. ferridurans*) [8, 9]. Comparative genomics reveals that some metal resistance genes of *Acidithiobacillus* spp. are acquired from microbial individuals who share the same habitat through early horizontal gene transfer (HGT) [10]. Additionally, the HGT elements of acidophiles are critical for their adaptive evolution in natural ecosystems, such as insertion sequence (IS), genomic island (GI) and prophage [11, 12].

IS has been recognized as the simplest type of mobile genetic elements (MGEs) and frequently identified in bacterial genomes [13]. Most ISs are between 700 and 2,500 bp in size and contain one or more open reading frames (ORFs) [14]. ISs consist of sequences that encode the transposase proteins in the middle and inverted repeats (IRs) at both ends. IRs act as recognition and cleavage sites for transposases during transposition process [15]. They usually generate flanking direct repeats (DRs) of the target DNA at the insertion site, and the DR length ranges from 2 to 14 bp approximately [16]. Transposase proteins determine the catalytic mechanisms for IS transposition. Depending on the catalytic mechanisms, ISs could be classified as DDE transposases, DEDD transposases, HUH transposases and serine transposases [17]. As the most abundant in bacteria and archaea, DDE transposases have three highly conserved acidic amino acid residues, namely aspartate (D), D and glutamate (E), in the critical catalytic motif [18, 19]. According to the overall genetic organization and specific genetic signatures, ISs also could be classified into different families (https://www-is.biotoul.fr/). The IS members within a family are assumed to employ the same or similar transposition mechanism, while the ISs within a family bear little sequence divergence [15, 20]. The active transposition accounts for the proliferation of IS copy and the two identical IS members have most likely arisen through transposition [14]. Insertion of ISs could interrupt genes and promoters, or affect transcriptional signals, thereby regulating the expression of surrounding genes. If the ISs translocated to the coding region, the mutation or frameshift may probably result in gene inactivation [21]. On the other hand, the insertion phenomenon may help bacteria recruit new metabolic pathways through large-scale genetic recombination and rearrangements. IS could also serve as a promoter for neighboring genes, driving their transcription [22].

Many researchers have focused on the comparative analysis of ISs within multiple genomes, emphasizing the vital roles of ISs for microbes to accommodate surrounding changes [10, 15]. For example, ISs from 262 prokaryotic genomes, including bacteria and archaea, have been surveyed [23]. Most genomes are found to have no more than 60 IS elements, while some have over 300 IS elements [23]. Meantime, the distribution of IS families in terms of family members and copy numbers in prokaryotic genomes is heterogeneous, which highlights their significant contribution to genome plasticity [23]. An inquiry into the presence, diversity and recent activity of ISs in *Thermus* spp. also reveals their importance for the biological adaptation of these thermophilic bacteria [24]. *Acidiphilium* carries various abilities by ISs and other MGEs, such as metal resistance and organic compound metabolism, facilitating beneficial interactions with other cohabitant autotrophs [25]. After analyzing the distribution and evolutionary dynamics of ISs within *Shigella* genomes, five ISs (IS1, IS2, IS4, IS600 and IS911) are found to undergo great expansion in all genomes, important for the evolutionary convergence and metabolic streamlining [26]. It has been confirmed that IS translocation could influence target sites, uptake determinants or the regulatory pathways; hence bacterial resistance to antimicrobial/xenobiotic increases [15, 27]. Therefore, ISs potentially drive genetic variation and enhance bacterial adaptation when counteracting environmental stress [28].

As a model system for the survey of extreme acidophiles, AMD provides a suitable environment for the deep exploration of bacterial ISs. It has been demonstrated that *Acidithiobacillus* absorbs and integrates additional novel functions through HGT, gene duplication and natural selection to accommodate the challenging surroundings [29]. Previous experiments using southern hybridization have confirmed the presence of ISAfe600, ISAfe1 and IST2 with multiple copies in five *A. ferrooxidans* genomes. Nevertheless, insertion of ISAfe1 into the *resB* gene eliminated the ability of *A. ferrooxidans* to oxidize Fe^2+^ [30, 31]. In addition, *A. ferridurans* lost its capacity to oxidize iron under high salt conditions, because the IS4 family transposition prevents the transcription of related genes but gains the tolerance to high salinity. When the bacteria are transferred to normal conditions, the IS66 family transposes, allowing it to oxidize iron again [32]. With the rapid advances in sequencing technology, more and more genome sequences of microorganisms in extremely acidic environment have been accessed. In the present study, the distribution properties and evolutionary relationships of ISs in 36 *Acidithiobacillus* genomes were systematically analyzed. The potential functions of the surrounding genes were annotated to reveal the possible gene regulation strategies based on ISs. These evidences will provide essential information to reveal the genomic plasticity and adaptive evolution of *Acidithiobacillus* in extremely acidic and oligotrophic environment.

## Materials and methods

### Genomes downloading

The genomes of 36 strains affiliated with the genus *Acidithiobacillus* were downloaded from the NCBI website (http://www.ncbi.nlm.nih.gov). They were *A. ferrooxidans* (9), *A. ferrivorans* (6), *A. thiooxidans* (11), *A. caldu*s (7), *A. albertensis* (1), *A. ferridurans* (1) and *Acidithiobacillus* sp. ‘AMD consortium’ (1). The information about general features of these 36 sequenced genomes were collected for further analysis.

### Identification, classification of the ISs and confirmation of IRs and DRs

The ISs and transposases within 36 genomes were predicted and classified based on the ISfinder platform (https://www-is.biotoul.fr/) using online BLASTN analysis with an *E*-value of 1e^− 10^ and the identity > 78% [33]. The ClustalW multiple alignment algorithm and Markov Cluster Algorithm (MCL) were employed to validate the classification of IS family and subgroup. The information about the position, family and length of all the ISs were obtained. Then the IS family, family members and copy numbers of each strain were calculated.

Both ends of ISs were also identified and defined. The DNA of transposases combined with 1000 bp upstream and downstream regions were extracted and examined manually to decern the IRs and flanking DRs. When more than a single IS copy was identified, BLASTN was used to define the IS ends. The ends were defined by identifying and comparing with empty sites when there was only a single copy [33, 34].

### Evolutionary analysis of the transposases within selected ISs and the host strains

Taking into account the ISs with more family members and copy numbers, representative IS families of *A. ferrooxidans*, *A. ferrivorans*, *A. thiooxidans* and *A. caldus* were chosen for phylogenetic analysis, respectively. The amino acid sequences of DDE transposases within selected ISs of each strain were aligned using ClustalW [35]. By employing MEGAX software, the phylogenetic trees were constructed via maximum likelihood algorithms with a bootstrap check of 5,000 replications [36]. Additionally, the coding protein sequences of four main species were predicted by Prokaryotic Dynamic Programming Genefinding Algorithm (Prodigal) [37]. Then the phylogenetic trees were constructed by CVTree4 [38] and the K-value was set at 6.

### Genetic mapping and sequence alignment of the ISs

The genome annotation was performed utilizing the Rapid Annotation Subsystem Technology (RAST) server (https://rast.nmpdr.org) [39]. The presence and components of the proximal regions around ISs were also obtained. Functional regions were compared and selected to generate physical gene maps, which were mainly around Tn3 and IS110. Furthermore, the nucleotide sequences of Tn3 family ISs were visualized using WebLogo (http://weblogo.threeplusone.com/) to find conserved regions. The amino acid sequences of transposases of IS110 were aligned together with RuvC (accession no. P24239) to show key residues by Clustal Omega [40].

### Statistical analysis

Linear regression analysis between the size, GC% of each genome and their total number of IS members as well as copy number of ISs were carried out using the R packages ggplot2 (v3.3.5) and ggpubr (v0.4.0).

## Results

### Overview of the IS family distribution in Acidithiobacillus genomes

**Fig. 1:**
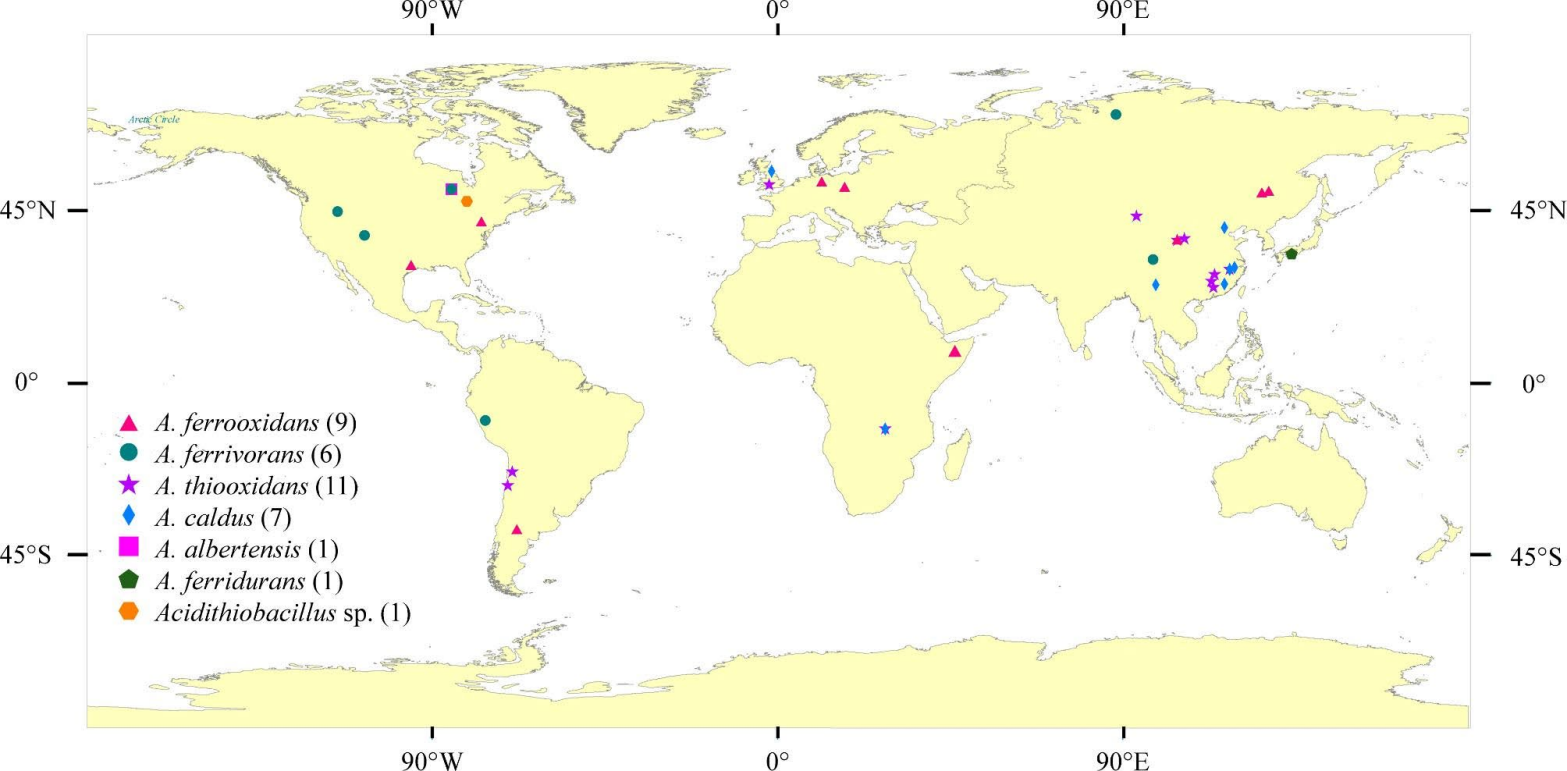
Geographical location of the 36 strains within the genus *Acidithiobacillus*

The main sequencing features (e.g. genome size, GC%) of 36 *Acidithiobacillus* strains were summarized (Table S1) and their geographical locations were exhibited in Fig. 1. In general, they were globally representative and mostly isolated from the AMD environments with the optimum pH lower than 3.

In all, 248 members belonging to 23 IS families with a total of 10,652 copies were identified within *Acidithiobacillus* genomes, which was listed in Table S2 and Table S3. Correlation analysis showed that the size and GC% of each genome were both not relevant to their total number of IS members (Fig. S1a, Fig. S1c). In contrast, the former and the latter significantly had a positive and negative association with the total copy number of ISs, respectively (Fig. S1b, Fig. S1d).

**Fig. 2:**
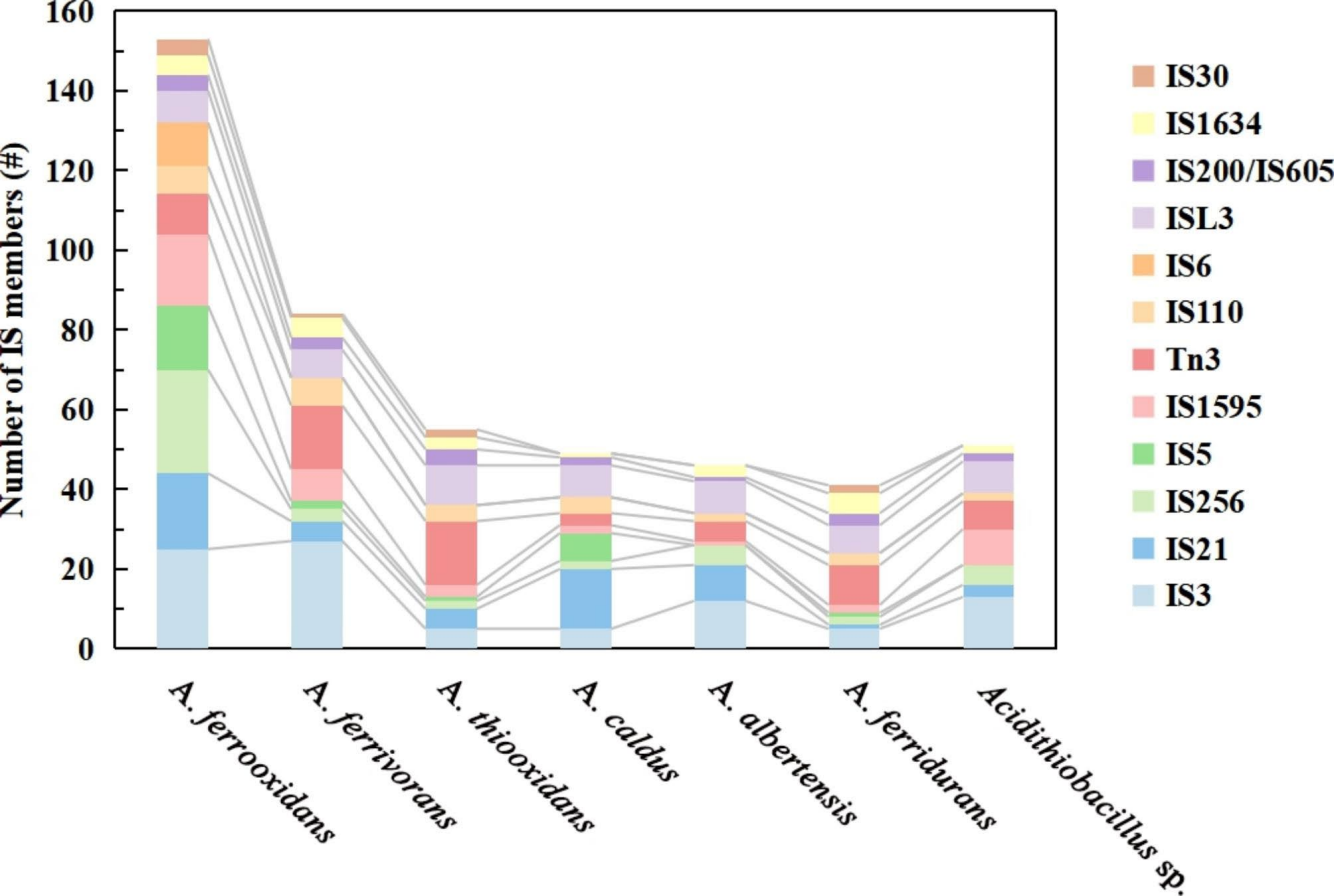
The distribution of IS members (top 12) in each *Acidithiobacillus* species

Concretely, *A. ferrooxidans* possessed the highest, 166 IS members, yet even there was only one strain within *A. ferridurans* and *A. albertensis*, they both had nearly 50 members (Table S3). The top 12 family with at least 4 families was shown in Fig. 2. The top five families, IS3, IS21, IS256, IS5 and IS1595, distributed over 60% of 36 genomes. By comparison, IS1380 was merely harbored by three strains. Interestingly, IS3, IS1595, IS110 and ISL3 presented in all the strains. IS256 and Tn3 were also detected in these strains except *A. caldus* ATCC 51756. On the contrary, IS6 with 11 members was exclusive for *A. ferrooxidans* DLC-5. Besides, the IS family distribution was non-uniform at the species level. For example, IS1634 and IS66 had a strong preferential insertion in iron and sulfur oxidizers *A. ferrooxidans* and *A. ferrivorans*, but they were rarely observed in sulfur-oxidizing *A. caldus*. Similarly, IS30 was prevalent in *A. thiooxidans*, but it was comparatively uncommon within *A. caldus* genomes. In contrast, the number of IS21 family members in each *A. caldus* was over 10, which in most *A. ferrivorans*, *A. thiooxidans* and *A. albertensis* was no more than 3. IS630 family was commonly detected in *A. thiooxidans* and IS1380 was the peculiar one to *A. caldus*, which were rarely identified in other species. The presence of IS families in *A. ferridurans* JCM 18981 and *Acidithiobacillus* sp. ‘AMD consortium’ was similar, but differently the latter strain was found to maintain the IS481.

### Evolutionary analysis of ISs in *Acidithiobacillus* genomes

The number of IS member and their copies in four main species were taken into account to pick appropriate ISs for phylogenetic analysis. *A. thiooxidans* genomes owned a maximum of 4,902 IS copies, while *A. ferrivorans* had 1,057 copies (Fig. S2). In *A. ferrooxidans*, IS3 with the most family members and copies was dominant in each strain (Fig. S3a, Fig. S4a). IS3 and Tn3 were predominant in *A. ferrivorans*, while Tn3 was superior with more total copy amounts (Fig. S3b, Fig. S4b). Tn3 and IS21 family with multiple copies contained the most family members in *A. thiooxidans* (Fig. S3c) and *A. caldus* (Fig. S3d), respectively, though ISL3 obviously maintained the highest copy number in each strain (Fig. S4c, S4d). Therefore, the representative IS family, which was the IS3 family of *A. ferrooxidans*, Tn3 family of *A. ferrivorans* and *A. thiooxidans*, and IS21 family of *A. caldus*, were selected for evolutionary analysis. These IS families belonged to typical DDE transposases, thus phylogenetic trees were constructed by using the amino acid sequences of DDE transposases located within ISs.

#### Phylogenetic analysis of IS3 family in *A. ferrooxidans*

**Fig. 3:**
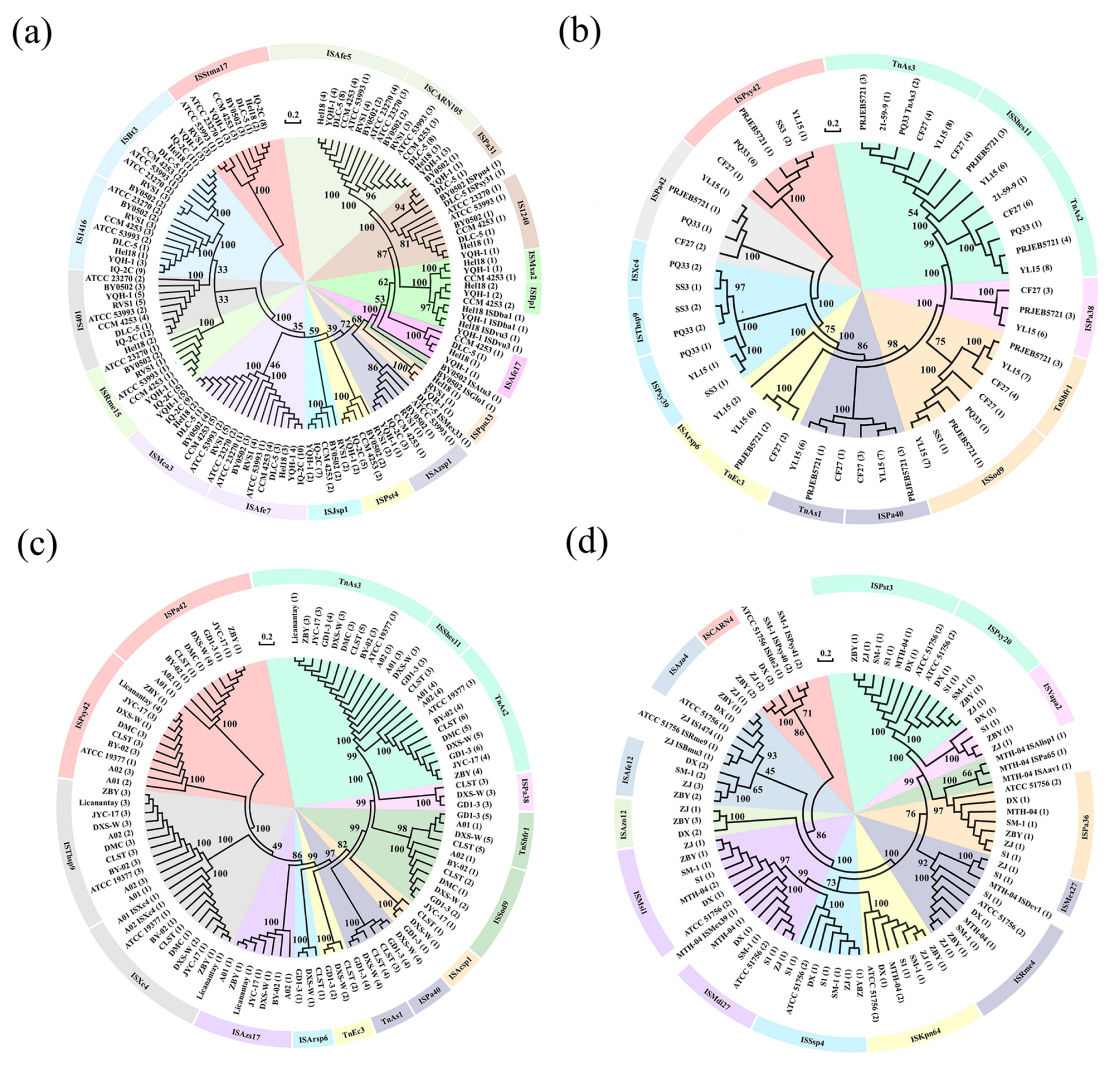
Evolutionary analysis of different ISs within *Acidithiobacillus* based on amino acid sequences. Phylogenetic tree of IS3 family within *A. ferrooxidans* strains (a). Phylogenetic tree of Tn3 family within *A. ferrivorans* strains (b). Phylogenetic analysis of Tn3 family within *A. thiooxidans* strains (c). Phylogenetic analysis of IS21 family within *A. caldus* strains (d)

The phylogenetic tree of 9 *A. ferrooxidans* genomes showed that they were apparently divided into two main groups and other clades with one single strain. Strains DLC-5, IO-2C and BY0502 were closely related to each other, as well as RVS1, CCM 4253, Hel18 and YQH-1 (Fig. S5a). Some IS3 family members were present in each *A. ferrooxidans*, such as ISStma17, ISBt3, IS1416, IS401, ISMca3 and ISAfe7 (Table S3). They were generally grouped respectively according to the classification of IS members rather than microbial taxonomy, as shown in Fig. 3a. Several ISs from *A. ferrooxidans* Hel18, YQH-1 and CCM 4253 with closer phylogenetic distance, were always obviously clustered, which were ISAfe17, ISBp1 and ISMxa2, forming different branches based on IS members, respectively. Even for closely related strains, they differed in copy numbers for the same IS member. Strain IO-2C always maintained the highest IS copy amounts, such as 12 copies of IS401, 11 copies of ISBt3 and 9 copies of IS1416, whereas other strains had merely 1 or 2 copies for the most ISs.

#### Phylogenetic analysis of Tn3 family in *A. ferrivorans*

The 6 *A. ferrivorans* strains were classified into three evolutionary clades (Fig. S5b). Strain 21-59-9 represented a distinct clade at the bottom of the tree, while SS3, PQ33 and PRJEB5721, CF27, YL15 were grouped separately. No IS was observed to be shared by these 6 strains simultaneously. In most cases, the same ISs were detected within 3, 4, or 5 genomes. Among them, strains PRJEB5721, CF27 and YL15 always possessed the same Tn3 family members, e.g., TnEc3, TnAs1, ISPa40, TnShfr1, ISPa38, and ISShes11 (Table S3). Their ISShes11 and TnAs3 from 5 strains except for SS3 were clustered in the same branch, showing high degree of homology. The majority of ISs clustered together based on the family member classification (Fig. 3b). Additionally, strain YL15 exhibited the most IS copies, such as 8 copies of TnAs3, 8 copies of TnAs2, 7 copies of TnShfr1 and 7 copies of ISPa406, but other strains had no more than 3 copies.

#### Phylogenetic analysis of Tn3 family in *A. thiooxidans*

The 11 *A. thiooxidans* strains were divided into four distinct clades. Strains GD1-3, DXS-W, CLST, Licanantay, ATCC 19377 and A02 were clustered together, so did JYC-17, BY-02 and DMC (Fig. S5c). Even from different genomes, the same family members were always located in a branch of the evolutionary tree (Fig. 3c). It was worth noting that ISShes11 of 3 strains and TnAs3 from all 11 strains were grouped, showing close evolutionary affinities. Highly homologous GD1-3 and DXS-W as well as CLST always possessed some specific ISs, e.g., ISArsp6, TnEc3, TnAs1, ISPa40, ISAcsp1 and ISPa38, forming small clades respectively (Table S3). Additionally, the copy number of a few ISs in some strains reached 6, but it was no more than 3 at the most time.

#### Phylogenetic analysis of IS21 family in *A. caldus*

The 7 strains of *A. caldus* were classified into three evolutionary clusters. ATCC 51756 was the most distantly related one to the other strains, meanwhile, two major groups were composed of S1, SM-1, MTH-04 and ZJ, ZBY, DX, respectively (Fig. S5d). As shown in Fig. 3d, ISfMsi7, ISSsp4, ISKpn64, ISRme4, ISPa36, and ISPst3 were held by all strains above, evenly forming different IS branches of the evolutionary tree. Highly related DX, ZBY and ZJ strains, also had shared IS members, e.g., ISCARN4, ISAzo12, and ISAfe12, clustered closely, respectively. Notably, several ISs of *A. caldus* MTH-04 were clustered into a small clade, which were ISAlisp1, ISAav1 and ISPa65. Except that ISAfe12 and ISAzo12 had 3 copies, the rest ISs only owned 1 or 2 copies in each strain (Table S3).

### Functional analysis of IS proximity genes in *Acidithiobacillus*

By causing genome insertions, deletions and inversions, ISs could greatly impact genomic rearrangements [41]. After analyzing and comparing the functions of IS neighboring genes, the Tn3, IS110 family and other noteworthy families were chosen to decipher flanking genes mainly in type strains. The specific characterization of Tn3 and IS110 was listed in Table S4 and Table S5, which were mainly classified as DDE transposases and DEDD transposases, respectively.

#### Functional analysis of genes around Tn3 family in *Acidithiobacillus*

**Fig. 4:**
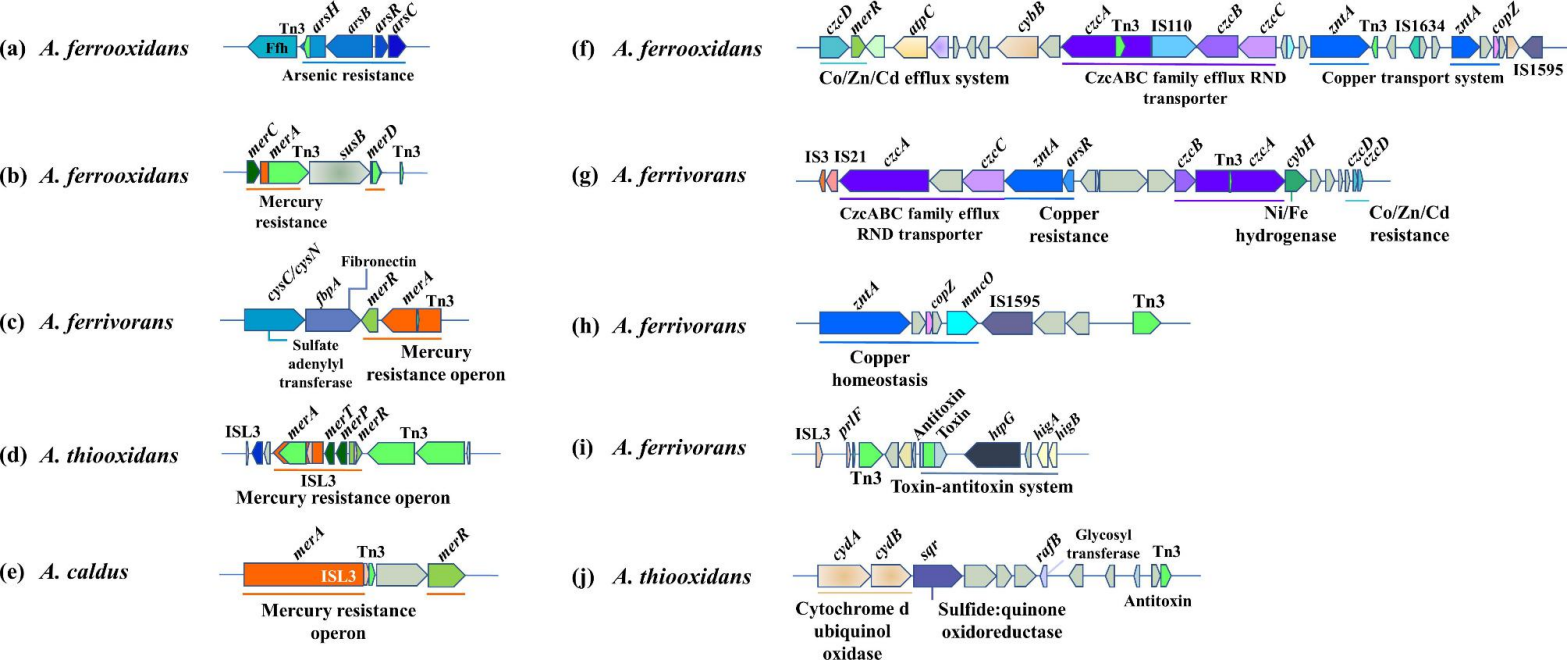
Putative laterally transferred regions contain ISs of Tn3 family within *Acidithiobacillus*. The representative strains in each species were selected as follows: (**a**) *A. ferrooxidans* ATCC 53993, (**b**) *A. ferrooxidans* ATCC 23270, (**c**) *A. ferrivorans* SS3, (**d**) *A. thiooxidans* DMC, (**e**) *A. caldus* ZBY, (**f**) *A. ferrooxidans* ATCC 23270, (**g**) *A. ferrivorans* PQ33, (**h**) *A. ferrivorans* SS3, (**i**) *A. ferrivorans* SS3, (**j**) *A. thiooxidans* ATCC 19377

The a*rsBRC* clusters encoding arsenic resistance was located near Tn3 in *A. ferrooxidans* ATCC 53993 (Fig. 4a). The main function of *arsBRC* genes is to reduce As(V) to As(III), and then pump As(III) out of the cell, thus *A. ferrooxidans* strains adapt to high concentrations of As(V) [10]. Many mercury resistant genes were identified around Tn3 in *A. ferrooxidans* ATCC 23270, *A. ferrivorans* SS3, *A. thiooxidans* DMC and *A. caldus* ZBY (Fig. 4b, c, d and e). The *czcD* gene coding Co/Zn/Cd resistance, *czcABC* cluster coding CzcABC family efflux RND transporter and *zntA* gene coding copper resistance were adjacent to Tn3 in *A. ferrooxidans* ATCC 23270 and *A. ferrivorans* PQ33 (Fig. 4f and g). The *czcD* was also close to IS1595 (Fig. S6a) and *czcB* was near ISL3 in *A. caldus* ATCC 51756 (Fig. S6b). Genes *zntA*, *copZ* and *mmcO* responsible for copper homeostasis were located upstream of IS1595 and Tn3 in *A. ferrivorans* SS3 (Fig. 4h). The *zntA* encodes the lead, cadmium, zinc, and mercury transporting ATPase, which pumps these metal ions from the cytoplasm to the periplasmic space [42]. The *copZ* gene encodes copper-translocating P-type ATPase that could pump Cu(I) ions from the cytosol to the periplasm [43]. *mmcO* encodes multicopper oxidase, oxidizing Cu(I) to less toxic form Cu(II) [44]. The toxin-antitoxin (TA) system was present downstream of Tn3 and ISL3 in *A. ferrivorans* SS3 (Fig. 4i), which was also found to be next to IS21 in *A. ferrooxidans* ATCC 23270 (Fig. S6c) and near IS3 in *A. caldus* ATCC 51756 (Fig. S6d). In *A. thiooxidans* ATCC 19377, *cydAB* (cytochrome d ubiquinol oxidase), *sqr* (sulfide: quinone oxidoreductase, SQR) and *rafB* (glycosyl transferase) genes appeared upstream of Tn3 (Fig. 4j). The cytochrome d ubiquinol oxidase is allowed for electron transfer as well as transmembrane proton pump activity [45]. Transposition within Tn3 family is often mediated by a large TnpA transposase with conversed recognition sequence [46], and our results reveled the similar BoxB2, BoxB1 and BoxA (Fig. S7a).

#### Functional analysis of genes around IS110 family in *Acidithiobacillus*

**Fig. 5:**
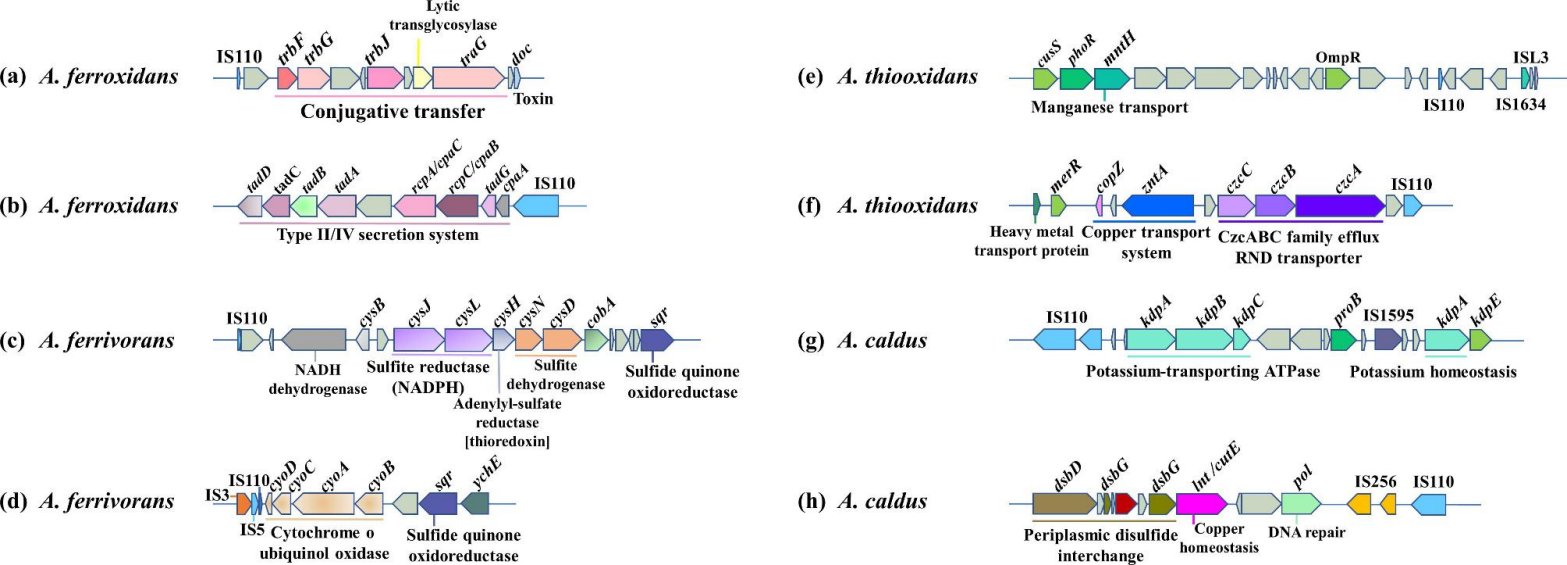
Putative laterally transferred regions contain ISs of IS110 family within *Acidithiobacillus*. The representative strains in each species were selected as follows: (**a**) *A. ferrooxidans* ATCC 23270, (**b**) *A. ferrooxidans* ATCC 23270, (**c**) *A. ferrivorans* SS3, (**d**) *A. ferrivorans* YL15, (**e**) *A. thiooxidans* ATCC 19377, (**f**) *A. thiooxidans* ATCC 19377, (**g**) *A. caldus* ATCC 51756, (**h**) *A. caldus* ATCC 51756

IS110 family had a tetrad D-E-D-D motif, corresponding to the catalytic center in the RuvC Holliday junction resolvase (Fig. S7b) [47]. Functional genes in proximity to IS110 were various. In *A. ferrooxidans* ATCC 23270, IS110 was present upstream of genes encoding conjugative transfer (Fig. 5a). These genes as well as the TA system were observed to be in the vicinity of IS1634, IS66 in *A. ferrooxidans* ATCC 53993 (Fig. S6e) and IS256 in *A. thiooxidans* ATCC 19377 (Fig. S6f). The conjugative transfer is an important way of transmitting genes between bacteria, for example, transferring plasmids and transposons could spread antibiotic resistance genes or other plasmid-borne genes to help bacteria to resist adverse environments [48, 49]. Meanwhile, genes coding type II/IV secretion system arranged upstream of IS110 in *A. ferrooxidans* ATCC 23270 (Fig. 5b) and downstream of IS5 in *A. caldus* SM-1 (Fig. S6g), which is undergone to deliver DNA and effector proteins into bacteria [50]. In *A. ferrivorans* SS3, IS110 was located upstream genes coding NADH dehydrogenase, sulfite reductase (NADPH), adenylyl-sulfate reductase (thioredoxin), sulfite dehydrogenase and SQR (Fig. 5c). Besides, IS110, IS3 and IS5 appeared upstream of *cyoABCD* genes encoding cytochrome o ubiquinol oxidase were capable of helping bacteria efficiently adapt to low oxygen conditions [51] and SQR in *A. ferrivorans* YL15 (Fig. 5d). In *A. thiooxidans* ATCC 19377, IS110 was present downstream of the *mntH* gene responsible for manganese transport (Fig. 5e) as well as *copZ*, *zntA* and *czcABC* clusters (Fig. 5f). In *A. caldus* ATCC 51756, *kdpABC* clusters coding potassium-transporting ATPase arranged upstream of IS110 (Fig. 5g). The cluster could pump potassium ions from the cytosol to the periplasm and *kdpE* controls the expression of *kdpABC* operon in response to potassium limitation or turgor pressure [52]. Additionally, genes encoding periplasmic disulfide interchange, copper homeostasis and DNA repair were found upstream of IS256 and IS110 (Fig. 5h). The *lnt/cutE* gene (copper homeostasis) was also identified near IS5 (Fig. S6h). The *cutE* acted as a copper storage gene that prevented copper from damaging intracellular constituents through free-radical reactions [53]. Besides, an immune system target invading DNA elements called type I restriction-modification (R-M) system coded by *hsdMSR* clusters attracted our attention. It lied downstream of IS21 in *A. ferrooxidans* ATCC 23270 (Fig. S6i), IS481 in *A. ferrivorans* CF27 (Fig. S6j) and *A. thiooxidans* ATCC 19377(Fig. S6k), or upstream of IS200/IS605, IS1595 in *A. caldus* ATCC 51756 (Fig. S6l).

## Discussion

Some bioinformatic and experimental surveys have focused on the presence and function of ISs in some *Acidithiobacillus* genomes. Previous work has demonstrated the presence of high similarity regarding IS families, IS1595, IS21 and IS3 in *A. ferrooxidans* and *A. ferridurans* [54], which is in accordance with our study. Southern blot analyses demonstrated that ISAfe1 belonging to ISL3 family exhibits transposition and may have been adapted to its host *A. ferrooxidans* [55]. Comparative genomic analyses of six *A. caldus* strains reveal that *A. caldus* ATCC 51756 and SM-1 acquire IS elements in the evolution course, resulting in higher genetic plasticity [56]. A large number of HGT events, for example ISs could help to increase the environmental adaptation of extremely acidophiles *Acidithiobacillus* [10].

Genome size is considered as the most relevant factor to the IS abundance, that is to say, the percentage of essential genes is lower in larger genomes, so ISs are more likely to result in a neutral or only slightly deleterious mutation [12, 23]. Our linear fitting analysis indicated that the larger the *Acidithiobacillus* genome, the more IS elements it would likely to contain. With the increase of GC percentage and genomic stability, it may be unfavorable for the ISs to replicate. The distribution of IS families and copy numbers of different species even within the same genus varied considerably, which were consistent with the previous study of 438 completely sequenced prokaryotic genomes [57], suggesting that the *Acidithiobacillus* species maintained differential genomic flexibility.

### The evolutionary relationships of ISs within *Acidithiobacillus*

IS with more copy amounts meant higher activity, otherwise, it cannot transpose easily [13, 23]. In this study, *A. thiooxidans* strains harbored the most IS copies, suggesting that their ISs were the most active within *Acidithiobacillus* and more likely to transpose. What’s more, *A. ferrooxidans* had the most diverse IS members, indicating that their genomes had developed more gene transposition strategies during the evolution process [23]. In *Escherichia coli*, the transposition activities of several IS families, Tn3, IS1, IS30 and IS911 remarkably declined at 42 ℃ [58]. The sensitivity of transposition to temperature has been regared as an inherent property for each transposase. For psychrotolerant *A. ferrivorans* and moderate thermophilic (45 °C optimum) *A. caldus* with relatively fewer IS members and copies, temperture of living conditions might be largly related to their transposition activities.

To discern the evolutionary relationships of ISs within *Acidithiobacillus*, typical IS families were selected from each *A. ferrooxidans*, *A. ferrivorans*, *A. thiooxidans* and *A. caldus* species based on IS distribution properties for phylogenetic analysis. No obvious clustering of ISs in the same strain except for *A. caldus* MTH-04 was observed, while the ISs belonging to the same member showcased a high degree of evolutionary relatedness even from different microbial individuals. In addition, ISShes11 and TnAs3 affiliated to Tn3 family may possess close affinity. ISs within a genome are usually extremely similar to each other and clustered in the same branch [59], thus it was assumed that the family IS3 from *A. ferrooxidans*, Tn3 from *A. ferrivorans* and *A. thiooxidans* and IS21 from *A. caldus* may have been in existence before the divergence of their host strains, respectively. That is to say, the co-evolve phenomenon between those ISs and their hosts was not witnessed [54, 60]. While for some homologous strains in each species, specific ISs were clustered according to the phylogeny of their host microbes. Those closely related strains may have developed IS-based strategies with similar evolutionary trends when facing natural stress. The copy numbers fluctuated in different genomes for the same IS member, such as *A. ferrooxidans* IO-2C and *A. ferrivorans* YL15, who exhibited the most copies of IS3 and Tn3 respectively, may have more transposition activities compared with other strains within their species. These results indicated that within *Acidithiobacillus* genomes, the recent activity of ISs was not solely determined by their own characteristics, but also depended on the host environment to a large extent.

### The environmental adaptation of *Acidithiobacillus* based on ISs

**Fig. 6:**
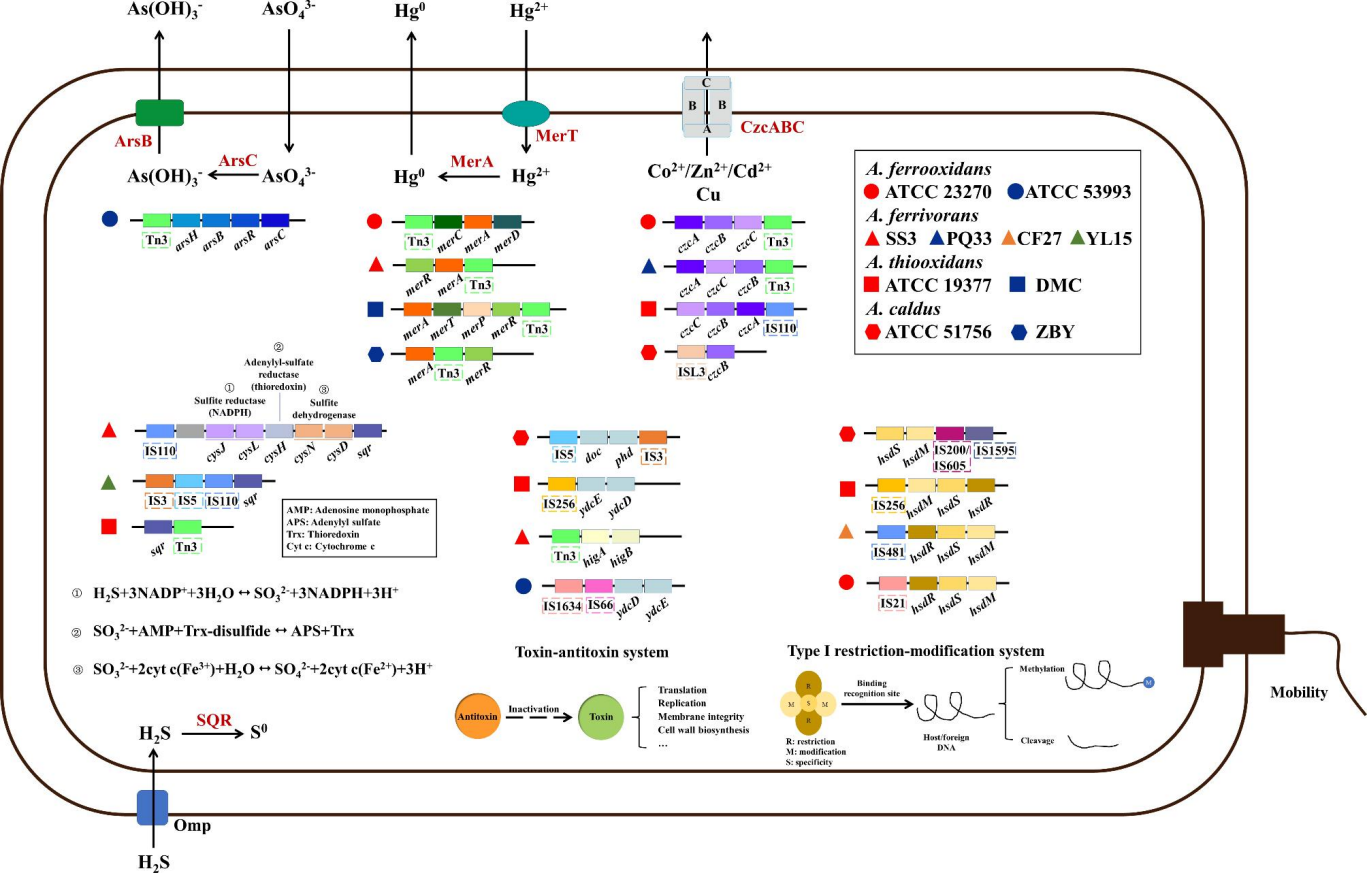
Schematic diagram showing the major metabolic pathways and adaptive mechanisms flanking some IS families in *Acidithiobacillus*

The extremely harsh acidic environment is often accompanied by high concentration of heavy metals and sulfate ions, throwing great challenges for the living microorganisms [29]. *Acidithiobacillus* had developed resistance to various metals based on ISs, which activate or silence the expression of related genes through transposition [8]. Tn3 family is ubiquitous in bacteria, shaping their host genomes through the paste-and-copy mechanism. Three conserved regions were indicted (Fig. S7a), in which the BoxA was relevant with DNA sequence recognition [46]. The Tn3 is featured by high degree of variability [61], which distributed near many heavy metal resistance regions. A schematic distribution of major adaptive mechanisms flanking some IS families in *Acidithiobacillus* were concluded (Fig. 6). For instance, the *merA* gene harbored by four *Acidithiobacillus* species in this study, could reduce Hg(II) to gaseous Hg(0) to decrease the local concentration of inorganic Hg(II). In *A. thiooxidans*, there was a unique *merT* gene encoding a transmembrane protein (MerT) that transports Hg(II) to the cytoplasm (Fig. 6). Additionally, the membrane efflux pump encoded by *czcABC* cluster is responsible for resistance to cadmium, zinc and cobalt in *Acidithiobacillus* [42]. The CzcABC family efflux RND transporter is a chemically permeable counter transport protein belonging to the RND superfamily [62]. It consists of CzcC (outer membrane protein), CzcB (coupling protein linking CzcC and CzcA) and CzcA (cytoplasmic membrane protein), forming a continuous channel linking the cytoplasm to the extracellular matrix [63]. There were some copper resistance genes such as *zntA*, *copZ*, *mmcO* in the vicinity of not only Tn3 but IS1634, IS1595 and IS5 in different species. Thus more than one HGT event may have occurred in the acquisition of these heavy metal resistance genes [10]. A large number of ISs translocated within functional regions regarding the heavy metal resistance of *Acidithiobacillus*, indicating that ISs especially Tn3 family may have participated in the expression of these genes and contributed to their survival in toxic metal-rich ecosystems [10]. In *Cupriavidus metallidurans* strains, transposition of ISRme5 and IS1086 provides promoters driving the transcription of *cnrCBAT*, thereby increasing the efficiency of zinc efflux and enhancing their adaptability to heavy metal-rich niches [64]. Additionally, the TA system acts on key cellular processes, including translation, replication, cytoskeleton formation, membrane integrity and cell wall biosynthesis, which was found to near Tn3 in *A. ferrivorans* (Fig. 6). When the antitoxin is not available under specific growth conditions and is rapidly degraded, the toxin is activated and acts on the cellular target site [65]. It has been reported that at least six different TA gene pairs are related to diverse Tn3 members. The investigation of the genetic context of TA systems suggests that they regulate to ensure stable invasion of Tn3 family transposons during transposition [66].

The IS110 is famous for DEDD transposases and is the only family defined not by transposase differentiation but the nature of IS terminus. The organization of its family members differs from that of the typical DDE ISs, which are related to the Holiday junction resolvase, RuvC [34]. The residues D, E, D, D were located in the catalytic center of the RuvC protein (Fig. S7b) [47]. Its insertion is not gene-specific but only related to the sequence of the insertion site thus the neighboring genes are diverse [19]. *Acidithiobacillus* has evolved multiple mechanisms of sulfur oxidation, utilizing sulfur-containing minerals to generate energy [67]. Sulfite reductase (NADPH) could catalyze the reduction of sulfite to sulfide [68]. Adenylyl-sulfate reductase (thioredoxin), using adenylyl-sulfate as a substrate and thioredoxin as an electron donor, reduces activated sulfate to sulfite [69]. Sulfite dehydrogenase catalyzes the oxidation of toxic and mutagenic compounded sulfite to sulfate. In this way, cells are protected from the adverse effects associated with sulfite exposure [70, 71]. SQR plays a crucial role in sulfide detoxification, energy production and sulfide homeostasis by supplying electrons to the respiratory or photosynthetic electron transport chain [72]. The above functional genes were discovered in proximity to IS110 (Fig. 6), which might get involved in regulating various sulfur oxidation mechanisms. By southern blot experiments, ISAfe1 belonging to IS110 family identified in *A. ferrooxidans* TFBk and TFO strains have been proven to exhibit transposable activity in the process of adapting to elemental sulfur [31]. What’s more, R-M enzymes are complexes that identify specific DNA sequences and modify them, usually by adding methyl groups, which serves to prevent the uptake of harmful or lethal DNA, such as lysing and killing bacteriophages (Fig. 6) [73]. IS5 and IS21 have been proven to facilitate the exchange of R-M gene cassettes between *Acidithiobacillus* and HGT vector [74]. Transposition insertional mutagenesis revealed that the ISAtc2 specifically inserted into *hsdM* gene, resulting in its inactivation [75]. On the contrast, IS6 family members such as Iso-ISS1 elements in *Lactococcus Lactis* Subsp. *lactis* could drive the expression of a R-M system responsible for phage resistance [76]. Analysis of the surrounding genes revealed that ISs had the potential to enhance resistance to heavy metals, help bacteria spread favorable genes, and increase sulfur oxidation potentials. Different ISs may have been involved in the evolutionary activation of functional adaptation of *Acidithiobacillus*, which remained to be demonstrated by manipulative experiments.

## Conclusions

Our results revealed significant variation in IS copy numbers and IS families within *Acidithiobacillus* genomes, suggesting the genomic flexibility. The evolutionary relationships of ISs were not consistent with that of their host genomes. Highly homologous ISs at the same branch on the phylogenetic tree differed in copy numbers in different genomes. Thus, the activity of ISs was determined not only by the characteristics of the IS themselves but also by the environmental pressures, which led these strains to evolve different IS-based strategies. Within *Acidithiobacillus* genomes, many ISs were located near the functional regions such as As/Hg/Cu/Co/Zn/Cd translocation, sulfur oxidation, and conjugative transfer, suggesting that ISs potentially affected their resistance to heavy metals and improved the adaptation of *Acidithiobacillus* to extremely acidic environment. This study has contributed to unravelling the evolutionary impact of ISs and functional differentiation of flanking genes in *Acidithiobacillus*, laying the foundation for further investigation of their genomic plasticity and adaptative evolution.

## Electronic supplementary material

Below is the link to the electronic supplementary material.

Supplementary Material 1

Supplementary Material 2

## Data Availability

All data generated or analyzed during this study are included in this published article and its supplementary information files. The genome sequences of strains in this study were downloaded from NCBI website (http://www.ncbi.nlm.nih.gov) and the accession number was listed in the Supplementary Table 1.

## Abbreviations

AMD: Acid mine drainage
HGT: Horizontal gene transfer
IS: Insertion sequence
GI: Genomic island
MGEs: Mobile genetic elements
ORFs: Open reading frames
IRs: Inverted repeats
DR: Direct repeats
D: Aspartate
E: Glutamate
MCL: Markov cluster algorithm
Prodigal: Prokaryotic dynamic programming genefinding algorithm
RAST: Rapid annotation subsystem technology
TA: Toxin-antitoxin
SQR: Sulfide:quinone oxidoreductase
R-M: Restriction-modification.

## References

[1] Chen H, Xiao T, Ning Z, Li Q, Xiao E, Liu Y, Xiao Q, Lan X, Ma L, Lu F (2020). In-situ remediation of acid mine drainage from abandoned coal mine by filed pilot-scale passive treatment system: performance and response of microbial communities to low pH and elevated Fe. Bioresour Technol.

[2] Korzhenkov AA, Toshchakov SV, Bargiela R, Gibbard H, Ferrer M, Teplyuk AV, Jones DL, Kublanov IV, Golyshin PN, Golyshina OV (2019). Archaea dominate the microbial community in an ecosystem with low-to-moderate temperature and extreme acidity. Microbiome.

[3] Gallego S, Esbrí JM, Campos JA, Peco JD, Martin-Laurent F, Higueras P (2021). Microbial diversity and activity assessment in a 100-year-old lead mine. J Hazard Mater.

[4] Wang X, Ma L, Wu J, Xiao Y, Tao J, Liu X (2020). Effective bioleaching of low-grade copper ores: insights from microbial cross experiments. Bioresour Technol.

[5] Sriaporn C, Campbell KA, Van Kranendonk MJ, Handley KM (2021). Genomic adaptations enabling *Acidithiobacillus* distribution across wide-ranging hot spring temperatures and pHs. Microbiome.

[6] Zhang X, Liu X, Yang F, Chen L (2018). Pan-genome analysis links the hereditary variation of *Leptospirillum ferriphilum* with its evolutionary adaptation. Front Microbiol.

[7] Zhang X, Liu X, Liang Y, Guo X, Xiao Y, Ma L, Miao B, Liu H, Peng D, Huang W, Zhang Y, Yin H. Adaptive evolution of extreme acidophile *Sulfobacillus thermosulfidooxidans* potentially driven by horizontal gene transfer and gene loss. Appl Environ Microbiol. 2017;83(7). 10.1128/AEM.03098-16 PMC5359484 28115381

[8] Zhang X, Liu X, Li L, Wei G, Zhang D, Liang Y, Miao B (2019). Phylogeny, divergent evolution, and speciation of sulfur-oxidizing *Acidithiobacillus* populations. BMC Genomics.

[9] Ma L, Huang S, Wu P, Xiong J, Wang H, Liao H, Liu X (2021). The interaction of acidophiles driving community functional responses to the re-inoculated chalcopyrite bioleaching process. Sci Total Environ.

[10] Li L, Liu Z, Meng D, Liu X, Li X, Zhang M, Tao J, Gu Y, Zhong S, Yin H (2019). Comparative genomic analysis reveals the distribution, organization, and evolution of metal resistance genes in the genus *Acidithiobacillus*. Appl Environ Microbiol.

[11] Guo J, Wang Q, Wang X, Wang F, Yao J, Zhu H (2015). Horizontal gene transfer in an acid mine drainage microbial community. BMC Genomics.

[12] Nelson WC, Wollerman L, Bhaya D, Heidelberg JF (2011). Analysis of insertion sequences in thermophilic cyanobacteria: exploring the mechanisms of establishing, maintaining, and withstanding high insertion sequence abundance. Appl Environ Microbiol.

[13] Consuegra J, Gaffé J, Lenski RE, Hindré T, Barrick JE, Tenaillon O, Schneider D (2021). Insertion-sequence-mediated mutations both promote and constrain evolvability during a long-term experiment with bacteria. Nat Commun.

[14] Wagner A, Lewis C, Bichsel M (2007). A survey of bacterial insertion sequences using IScan. Nucleic Acids Res.

[15] Vandecraen J, Chandler M, Aertsen A, Van Houdt R (2017). The impact of insertion sequences on bacterial genome plasticity and adaptability. Crit Rev Microbiol.

[16] Zhou F, Olman V, Xu Y (2008). Insertion sequences show diverse recent activities in Cyanobacteria and Archaea. BMC Genomics.

[17] Sadler M, M RM. R LF. Characterization of the IS200/IS605 insertion sequence family in *Halanaerobium Hydrogeniformans*. Genes (Basel). 2020;11(5). 10.3390/genes11050484 PMC7290912 32365520

[18] Guérillot R, Siguier P, Gourbeyre E, Chandler M, Glaser P (2014). The diversity of prokaryotic DDE transposases of the mutator superfamily, insertion specificity, and association with conjugation machineries. Genome Biol Evol.

[19] Siguier P, Gourbeyre E, Varani A, Ton-Hoang B, Chandler M (2015). Everyman’s guide to bacterial insertion sequences. Microbiol Spectr.

[20] He S, Hickman AB, Varani AM, Siguier P, Chandler M, Dekker JP, Dyda F, Davies JE (2015). Insertion sequence IS26 reorganizes plasmids in clinically isolated multidrug-resistant bacteria by replicative transposition. mBio.

[21] Arashida H, Odake H, Sugawara M, Noda R, Kakizaki K, Ohkubo S, Mitsui H, Sato S, Minamisawa K (2022). Evolution of rhizobial symbiosis islands through insertion sequence-mediated deletion and duplication. ISME J.

[22] Tempel S, Bedo J, Talla E (2022). From a large-scale genomic analysis of insertion sequences to insights into their regulatory roles in prokaryotes. BMC Genomics.

[23] Touchon M, Rocha EPC (2007). Causes of insertion sequences abundance in prokaryotic genomes. Mol Biol Evol.

[24] Blesa A, Sánchez M, Sacristán-Horcajada E, González-de la Fuente S, Peiró R, Berenguer J. Into the *Thermus* mobilome: Presence, diversity and recent activities of insertion sequences across *Thermus* spp. Microorganisms. 2019;7(1). 10.3390/microorganisms7010025 PMC6352166 30669685

[25] Li L, Liu Z, Zhang M, Meng D, Liu X, Wang P, Li X, Jiang Z, Zhong S, Jiang C (2020). Insights into the metabolism and evolution of the genus *Acidiphilium*, a typical acidophile in acid mine drainage. mSystems.

[26] Jane H, M MJ, Helen BJ, Bernhard P. E HK. Impact of insertion sequences on convergent evolution of Shigella species. PLoS genetics. 2020;16(7). 10.1371/journal.pgen.1008931 PMC7373316 32644999

[27] Razavi M, Kristiansson E, Flach CF, Larsson DGJ. The Association between insertion sequences and antibiotic resistance genes. mSphere. 2020;5(5). 10.1128/mSphere.00418-20 PMC7471000 32878926

[28] Adams MD, Bishop B, Wright MS (2016). Quantitative assessment of insertion sequence impact on bacterial genome architecture. Microb Genom.

[29] Navarro CA, von Bernath D, Jerez CA (2013). Heavy metal resistance strategies of acidophilic bacteria and their acquisition: importance for biomining and bioremediation. Biol Res.

[30] Kondrat’eva TF, Danilevich VN, Karavaiko GI (2008). The primary structure and characteristics of ISAfe600, an insertion sequence from *Acidithiobacillus ferrooxidans* strains. Microbiology.

[31] Kondrat’eva TF, Danilevich VN, Ageeva SN, Karavaiko GI (2005). Identification of IS elements in *Acidithiobacillus ferrooxidans* strains grown in a medium with ferrous iron or adapted to elemental sulfur. Arch Microbiol.

[32] Bonnefoy V, Grail BM, Johnson DB. Salt stress-induced loss of iron oxidoreduction activities and reacquisition of that phenotype depend on *rus* operon transcription in *Acidithiobacillus ferridurans*. Appl Environ Microbiol. 2018;84(7). 10.1128/AEM.02795-17 PMC5861818 29374029

[33] Siguier P, Perochon J, Lestrade L, Mahillon J, Chandler M (2006). ISfinder: the reference centre for bacterial insertion sequences. Nucleic Acids Res.

[34] Siguier P, Gourbeyre E, Chandler M (2014). Bacterial insertion sequences: their genomic impact and diversity. FEMS Microbiol Rev.

[35] Thompson JD, Gibson TJ, Higgins DG. Multiple sequence alignment using ClustalW and ClustalX. Curr Protoc Bioinformatics. 2003;00(1):2.3.1–2.3.22. 10.1002/0471250953.bi0203s00 18792934

[36] Kumar S, Stecher G, Li M, Knyaz C, Tamura K (2018). MEGA X: Molecular Evolutionary Genetics Analysis across Computing Platforms. Mol Biol Evol.

[37] Hyatt D, Chen GL, Locascio PF, Land ML, Larimer FW, Hauser LJ (2010). Prodigal: prokaryotic gene recognition and translation initiation site identification. BMC Bioinformatics.

[38] Zuo G, CVTree (2021). A parallel alignment-free phylogeny and taxonomy tool based on composition vectors of genomes. Genomics Proteom Bioinf.

[39] Overbeek R, Olson R, Pusch GD, Olsen GJ, Davis JJ, Disz T, Edwards RA, Gerdes S, Parrello B, Shukla M (2014). The SEED and the Rapid Annotation of microbial genomes using Subsystems Technology (RAST). Nucleic Acids Res.

[40] Sievers F, Wilm A, Dineen D, Gibson TJ, Karplus K, Li W, Lopez R, McWilliam H, Remmert M, Söding J (2011). Fast, scalable generation of high-quality protein multiple sequence alignments using Clustal Omega. Mol Syst Biol.

[41] Singh NK, Badet T, Abraham L, Croll D (2021). Rapid sequence evolution driven by transposable elements at a virulence locus in a fungal wheat pathogen. BMC Genomics.

[42] Mangold S, Potrykus J, Björn E, Lövgren L, Dopson M (2013). Extreme zinc tolerance in acidophilic microorganisms from the bacterial and archaeal domains. Extremophiles.

[43] Barahona S, Castro-Severyn J, Dorador C, Saavedra C, Remonsellez F. Determinants of copper resistance in *Acidithiobacillus ferrivorans* ACH isolated from the chilean altiplano. Genes (Basel). 2020;11(8). 10.3390/genes11080844 PMC7463520 32722087

[44] Kinkar E, Kinkar A, Saleh M (2019). The multicopper oxidase of *Mycobacterium tuberculosis* (MmcO) exhibits ferroxidase activity and scavenges reactive oxygen species in activated THP-1 cells. Int J Med Microbiol.

[45] Moya-Beltrán A, Beard S, Rojas-Villalobos C, Issotta F, Gallardo Y, Ulloa R, Giaveno A, Degli Esposti M, Johnson DB, Quatrini R (2021). Genomic evolution of the class Acidithiobacillia: deep-branching Proteobacteria living in extreme acidic conditions. ISME J.

[46] Shkumatov AV, Aryanpour N, Oger CA, Goossens G, Hallet BF, Efremov RG (2022). Structural insight into Tn3 family transposition mechanism. Nat Commun.

[47] Choi S, Ohta S, Ohtsubo E (2003). A novel IS element, IS621, of the IS110/IS492 family transposes to a specific site in repetitive extragenic palindromic sequences in Escherichia coli. J Bacteriol.

[48] Wang Q, Liu L, Hou Z, Wang L, Ma D, Yang G, Guo S, Luo J, Qi L, Luo Y (2020). Heavy metal copper accelerates the conjugative transfer of antibiotic resistance genes in freshwater microcosms. Sci Total Environ.

[49] Hall JPJ, Harrison E, Pärnänen K, Virta M, Brockhurst MA. The impact of mercury selection and conjugative genetic elements on community structure and resistance gene transfer. Front Microbiol. 2020;11(1846). 10.3389/fmicb.2020.01846 PMC7419628 32849443

[50] Escobar CA, Douzi B, Ball G, Barbat B, Alphonse S, Quinton L, Voulhoux R, Forest KT (2021). Structural interactions define assembly adapter function of a type II secretion system pseudopilin. Structure.

[51] Lunak ZR, Noel KD (2015). A quinol oxidase, encoded by *cyoABCD*, is utilized to adapt to lower O_2_ concentrations in Rhizobium etli CFN42. Microbiol (Reading).

[52] Sweet ME, Larsen C, Zhang X, Schlame M, Pedersen BP, Stokes DL (2021). Structural basis for potassium transport in prokaryotes by KdpFABC. Proc Natl Acad Sci U S A.

[53] Giachino A, Waldron KJ (2020). Copper tolerance in bacteria requires the activation of multiple accessory pathways. Mol Microbiol.

[54] Chen J, Liu Y, Diep P, Mahadevan R (2021). Genomic analysis of a newly isolated *Acidithiobacillus ferridurans* JAGS strain reveals its adaptation to acid mine drainage. Minerals.

[55] Holmes DS, Zhao HL, Levican G, Ratouchniak J, Bonnefoy V, Varela P, Jedlicki E (2001). ISAfe1, an ISL3 family insertion sequence from *Acidithiobacillus ferrooxidans* ATCC 19859. J Bacteriol.

[56] Zhang X, Liu X, He Q, Dong W, Zhang X, Fan F, Peng D, Huang W, Yin H. Gene turnover contributes to the evolutionary adaptation of *Acidithiobacillus caldus*: Insights from comparative genomics. Front Microbiol. 2016;7(1960). 10.3389/fmicb.2016.01960 PMC5138436 27999570

[57] Wagner A, de la Chaux N (2008). Distant horizontal gene transfer is rare for multiple families of prokaryotic insertion sequences. Mol Genet Genomics.

[58] Nagy Z, Chandler M (2004). Regulation of transposition in bacteria. Res Microbiol.

[59] Wagner A (2006). Periodic extinctions of transposable elements in bacterial lineages: evidence from intragenomic variation in multiple genomes. Mol Biol Evol.

[60] Ouahrani S, Michaux S, Widada JS, Bourg G, Tournebize R, Ramuz M, Liautard J-P (1993). Identification and sequence analysis of IS6501, an insertion sequence in *Brucella* spp.: relationship between genomic structure and the number of IS6501 copies. Microbiology.

[61] Nicolas E, Lambin M, Dandoy D, Galloy C, Nguyen N, Oger CA, Hallet B. The Tn3-family of replicative transposons. Microbiol Spectr. 2015;3(4). 10.1128/microbiolspec.MDNA3-0060-2014 26350313

[62] Alvarez-Ortega C, Olivares J, Martinez J. RND multidrug efflux pumps: what are they good for? Front Microbiol. 2013;4(7). 10.3389/fmicb.2013.00007 PMC3564043 23386844

[63] Chen M, Wang L, Zheng X, Cohen M, Li X (2021). Cross-kingdom comparative transcriptomics reveals conserved genetic modules in response to cadmium stress. mSystems.

[64] Vandecraen J, Monsieurs P, Mergeay M, Leys N, Aertsen A, Van Houdt R (2016). Zinc-induced transposition of insertion sequence elements contributes to increased adaptability of *Cupriavidus metallidurans*. Front Microbiol.

[65] Hayes F, Van Melderen L (2011). Toxins-antitoxins: diversity, evolution and function. Crit Rev Biochem Mol Biol.

[66] Lima-Mendez G, Oliveira Alvarenga D, Ross K, Hallet B, Van Melderen L, Varani AM, Chandler M. Toxin-antitoxin gene pairs found in Tn3 family transposons appear to be an integral part of the transposition module. mBio. 2020;11(2). 10.1128/mBio.00452-20 PMC7157771 32234815

[67] Zhang X, Liu X, Liang Y, Fan F, Zhang X, Yin H (2016). Metabolic diversity and adaptive mechanisms of iron- and/or sulfur-oxidizing autotrophic acidophiles in extremely acidic environments. Environ Microbiol Rep.

[68] Askenasy I, Murray DT, Andrews RM, Uversky VN, He H, Stroupe ME (2018). Structure–function relationships in the oligomeric NADPH-dependent assimilatory sulfite reductase. Biochemistry.

[69] Vigneron A, Cruaud P, Culley AI, Couture RM, Lovejoy C, Vincent WF (2021). Genomic evidence for sulfur intermediates as new biogeochemical hubs in a model aquatic microbial ecosystem. Microbiome.

[70] Low L, Kilmartin J, Bernhardt P, Kappler U (2011). How are “atypical” sulfite dehydrogenases linked to cell metabolism? Interactions between the SorT sulfite dehydrogenase and small redox proteins. Front Microbiol.

[71] Baker BJ, Lazar CS, Teske AP, Dick GJ (2015). Genomic resolution of linkages in carbon, nitrogen, and sulfur cycling among widespread estuary sediment bacteria. Microbiome.

[72] Zhang Y, Qadri A, Weiner JH (2016). The quinone-binding site of *Acidithiobacillus ferrooxidans* sulfide: Quinone oxidoreductase controls both sulfide oxidation and quinone reduction. Biochem Cell Biol.

[73] Waldron DE, Lindsay JA (2006). Sau1: a novel lineage-specific type I restriction-modification system that blocks horizontal gene transfer into *Staphylococcus aureus* and between *S. aureus* isolates of different lineages. J Bacteriol.

[74] Covarrubias PC, Moya-Beltrán A, Atavales J, Moya-Flores F, Tapia PS, Acuña LG, Spinelli S, Quatrini R (2018). Occurrence, integrity and functionality of AcaML1–like viruses infecting extreme acidophiles of the *Acidithiobacillus* species complex. Res Microbiol.

[75] Chen L, Lin J, Liu X, Pang X, Lin H, Lin J (2013). Transposition of IS elements induced by electroporation of suicide plasmid in *Acidithiobacillus caldus*. Enzyme Microb Technol.

[76] Vasu K, Nagaraja V (2013). Diverse functions of restriction-modification systems in addition to cellular defense. Microbiol Mol Biol Rev.

