# Supplementary material for: Insertion sequence contributes to the evolution and environmental adaptation of Acidithiobacillus
Source: BMC Genomics. 2023 May 25;24:282. doi: 10.1186/s12864-023-09372-8 (PMC10214660; doi:10.1186/s12864-023-09372-8)
Supplement: Supplementary file 2 — Supplementary Material 2 [file 12864_2023_9372_MOESM2_ESM.docx]

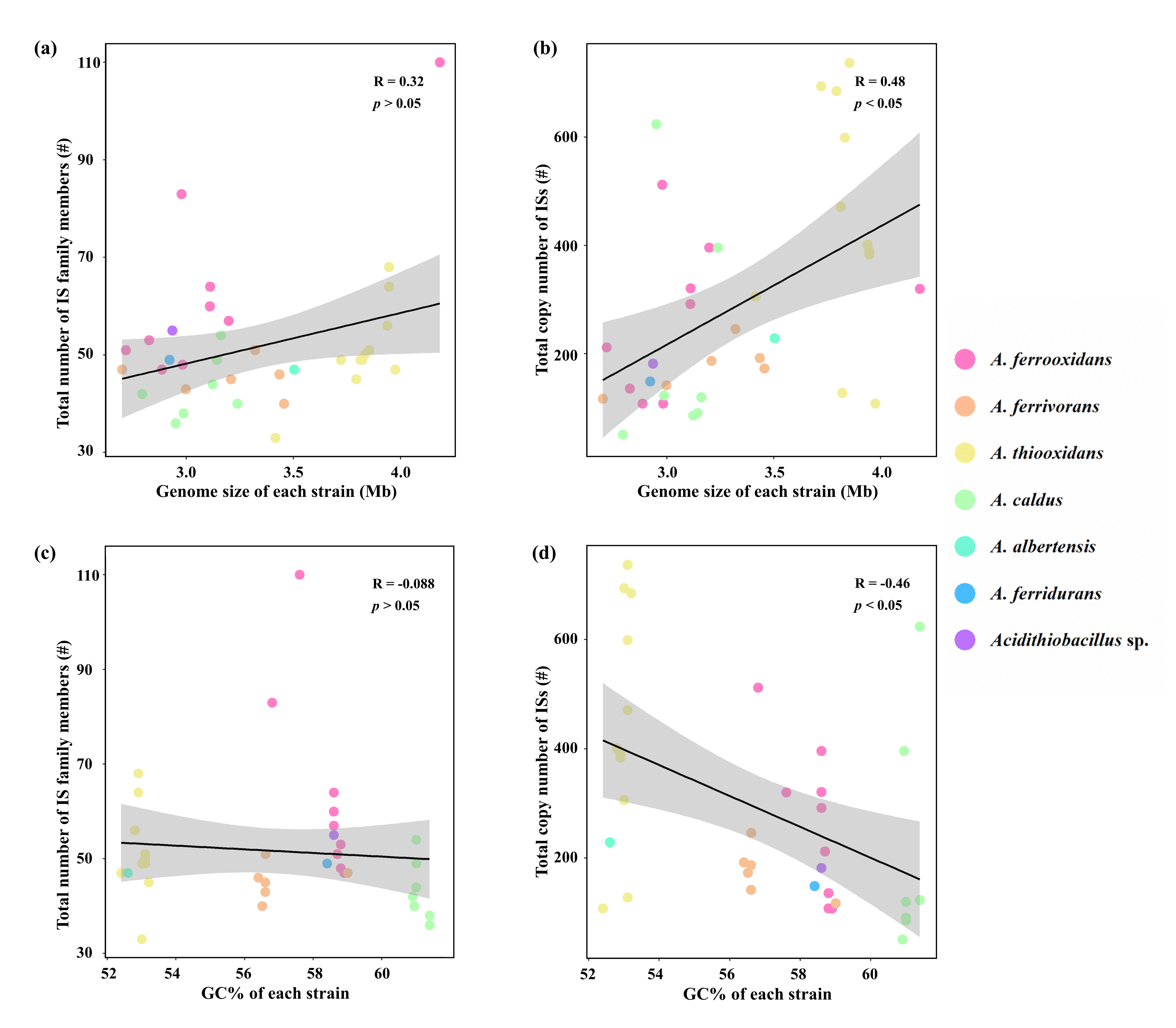


**Figure S1.** Relationship between genome size (Mb) of each *Acidithiobacillus* strain and total number of IS family members (a), total copy number of ISs (b), respectively. Relationship between GC% of each *Acidithiobacillus* strain and total number of IS family members (c), total copy number of ISs (d), respectively.


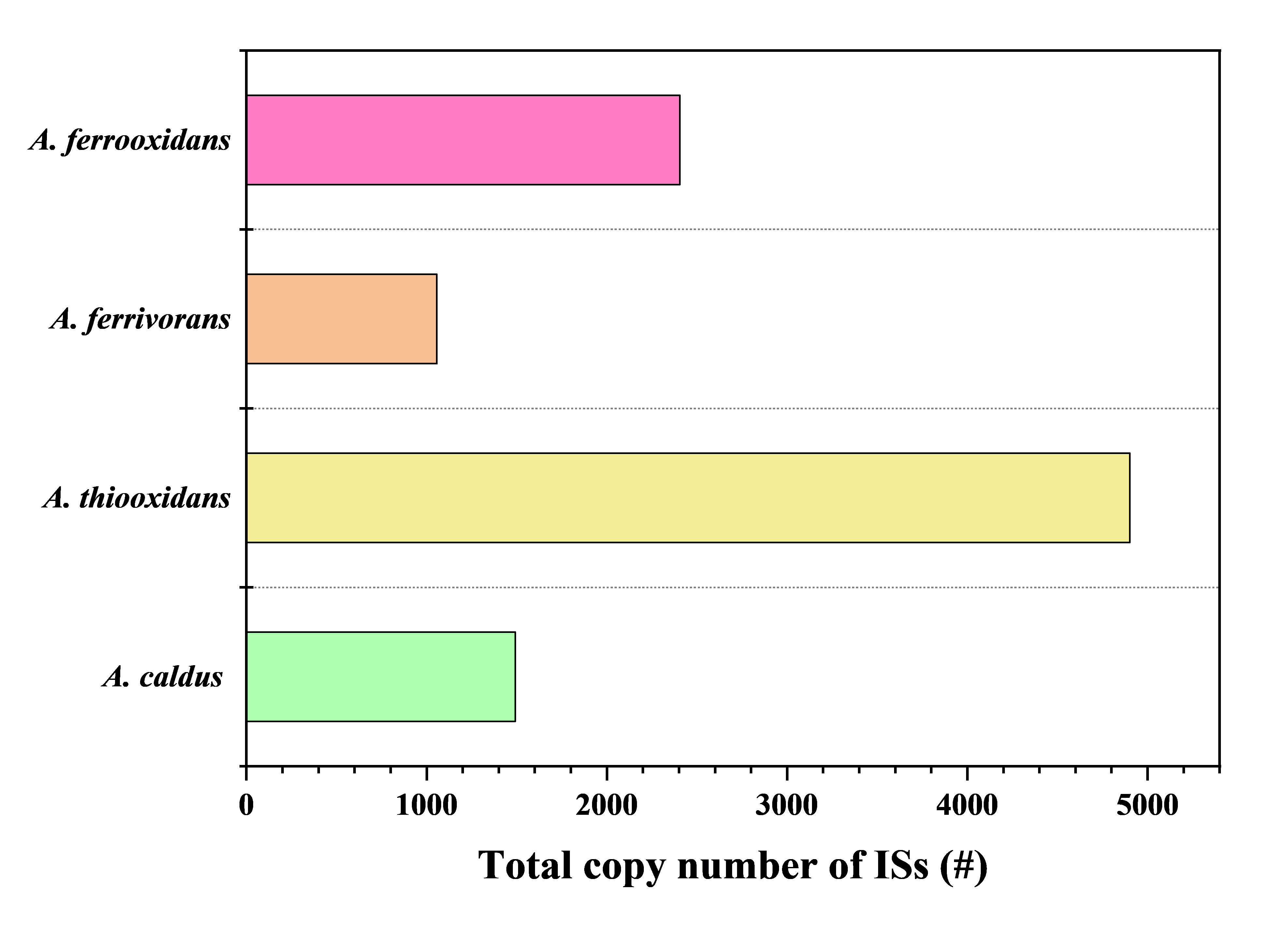


**Figure S2.** Total copy numbers of ISs in the four species of *Acidithiobacillus*.


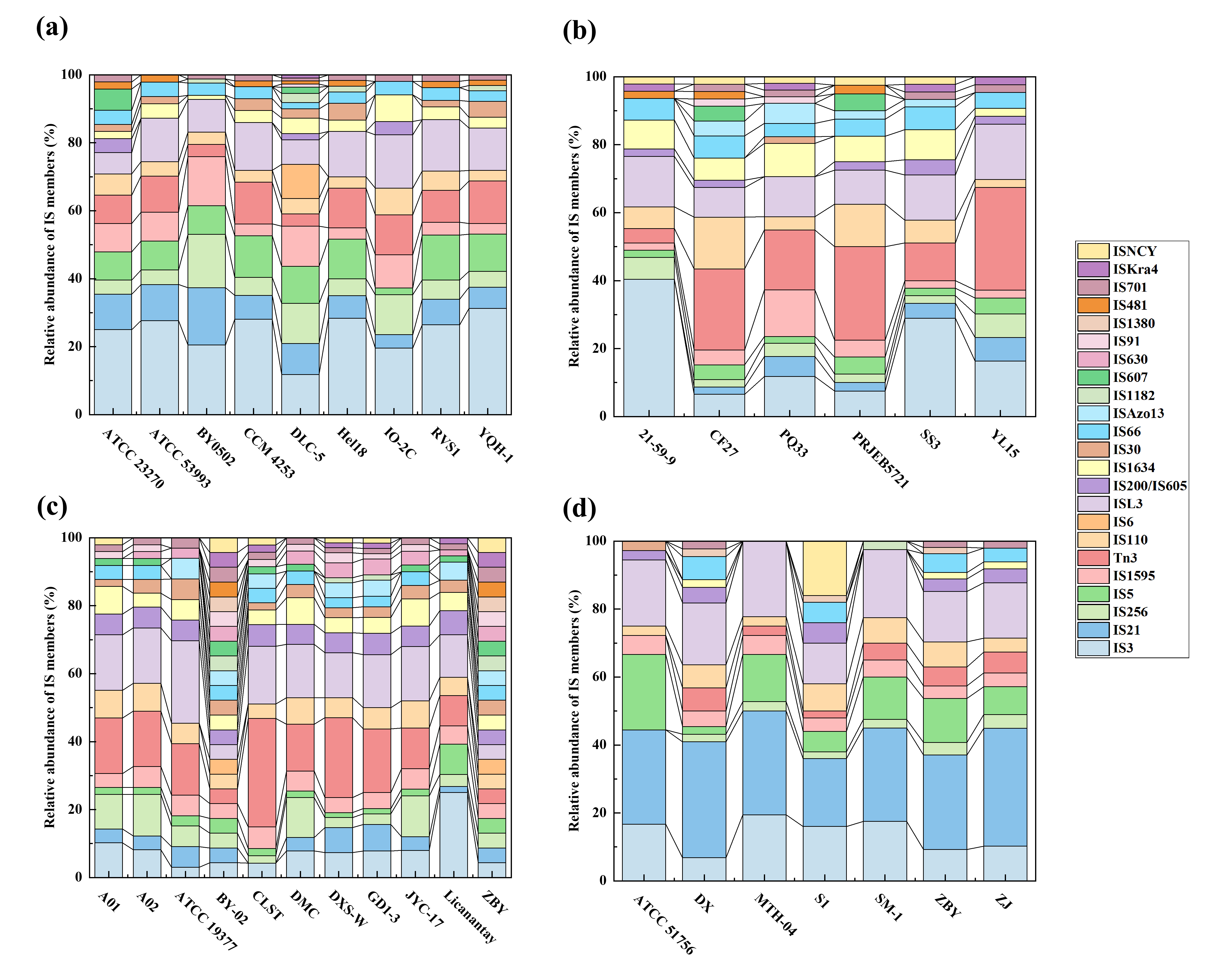


**Figure S3.** The number of IS family member in *A. ferrooxidans* (a), *A. ferrivorans* (b), *A. thiooxidans* (c), and *A. caldus* (d) strains.


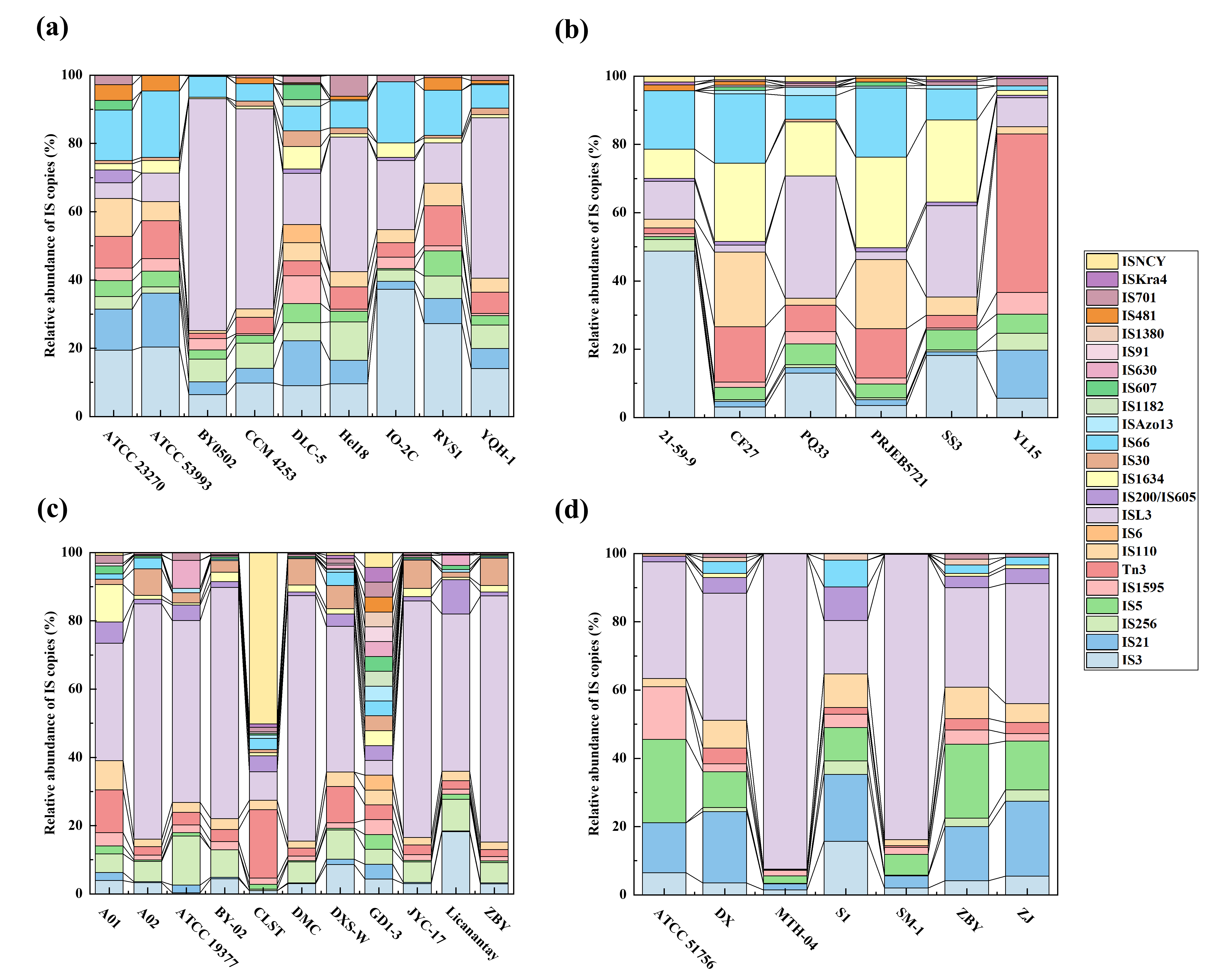


**Figure S4.** The copy number of IS family in *A. ferrooxidans* (a), *A. ferrivorans* (b), *A. thiooxidans* (c), and *A. caldus* (d) strains.


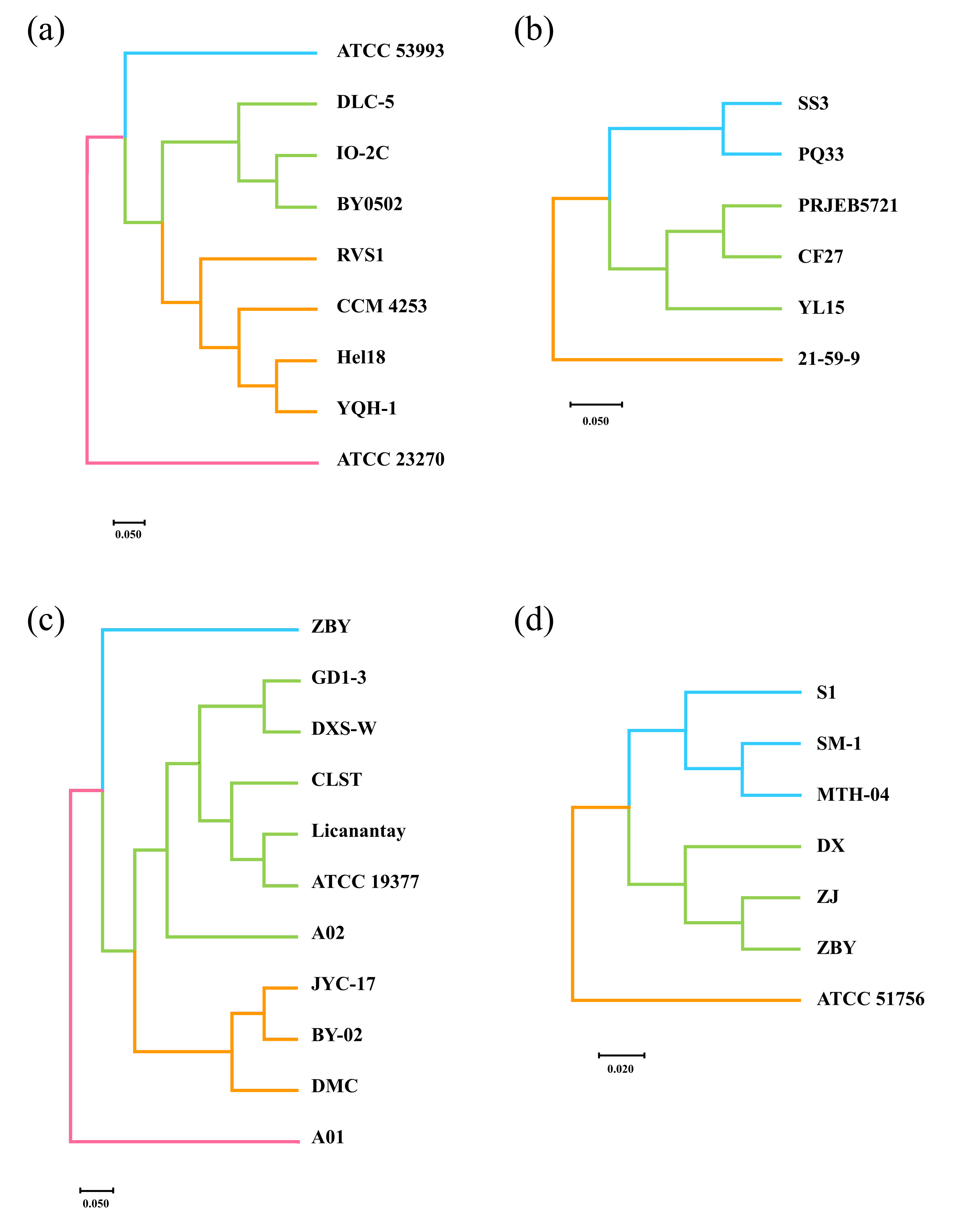


**Figure S5.** Phylogenetic analysis based on the coding protein sequences belonging with *A. ferrooxidans* (a), *A. ferrivorans* (b), *A. thiooxidans* (c), and *A. caldus* (d) strains.


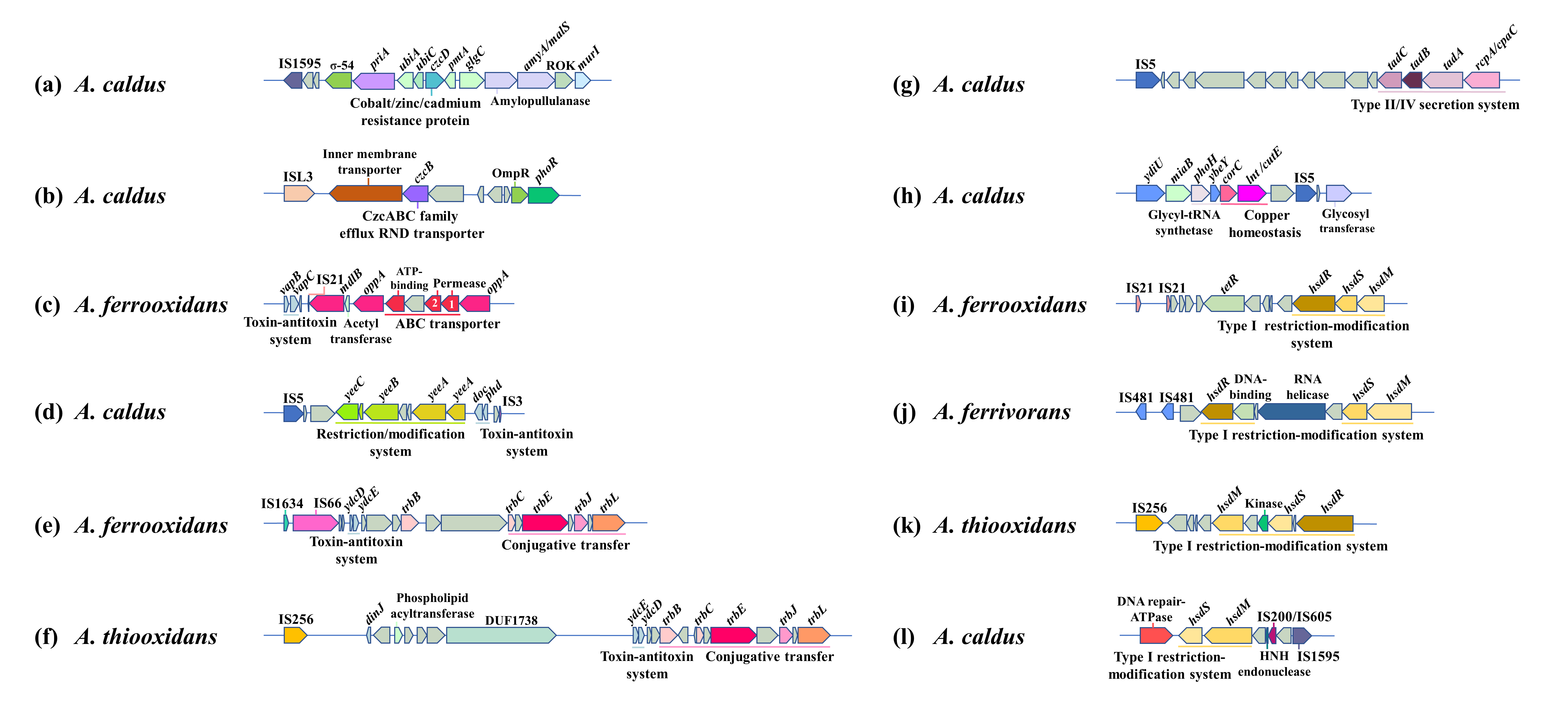


**Figure S6.** Putative laterally transferred regions contain ISs in other families within *Acidithiobacillus*. The representative strains in each species were selected as follows: (a) *A. caldus* ATCC 51756, (b) *A. caldus* ATCC 51756, (c) *A. ferriooxidans* ATCC 23270, (d) *A. caldus* ATCC 51756, (e) *A. ferriooxidans* ATCC 53993, (f) *A. thiooxidans* ATCC 19377, (g) *A. caldus* SM-1, (h) *A. caldus* ATCC 51756, (i) *A. ferriooxidans* ATCC 23270, (j) *A. ferrivorans* CF27, (k) *A. thiooxidans* ATCC 19377, (l) *A. caldus* ATCC 51756.


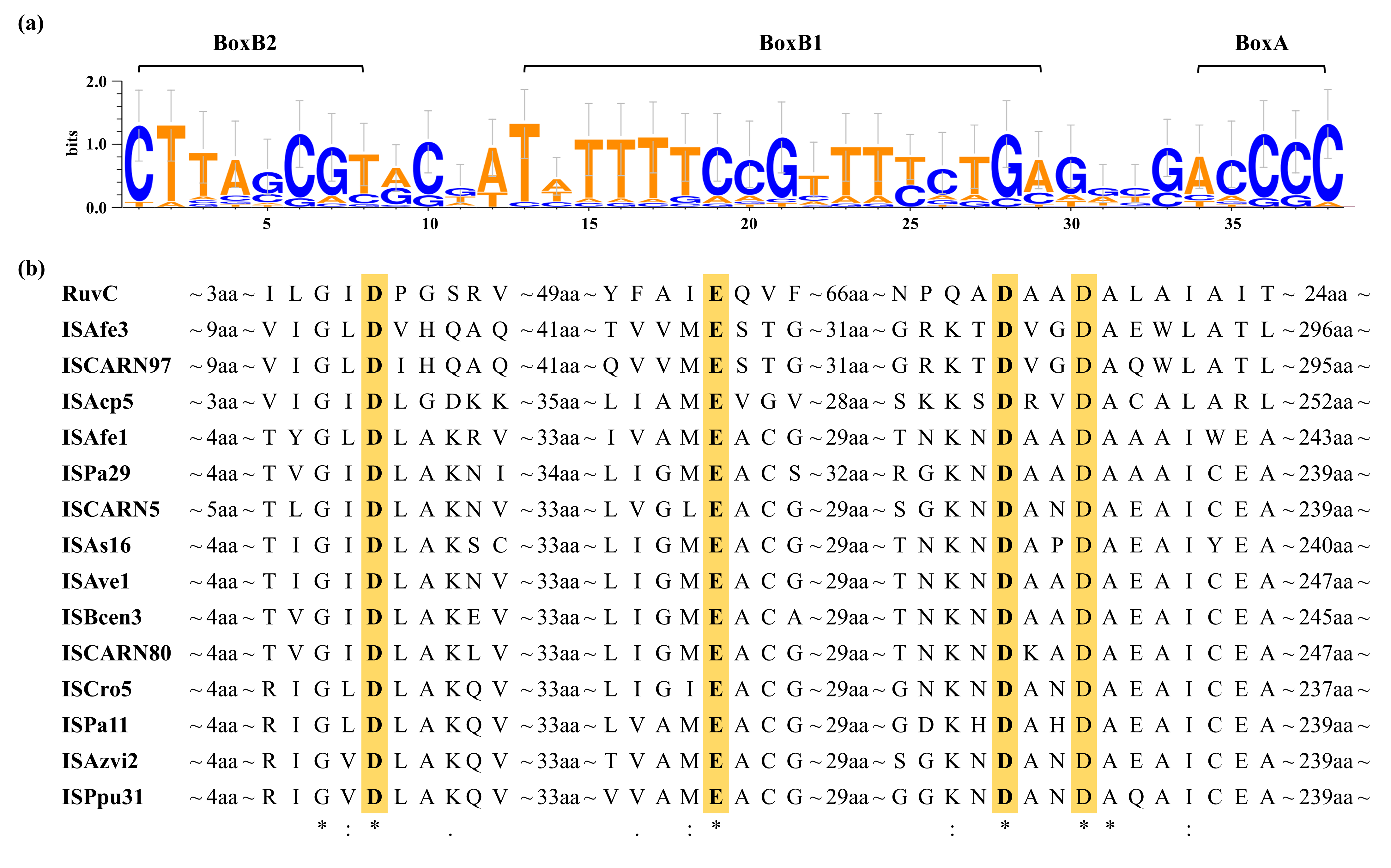


**Figure S7.** The conserved regions of Tn3 ISs (a) and the alignment of IS110 transposes with RuvC Holliday junction resolvase (accession no. P24239) (b).
